# The ionic liquid-assisted sample preparation method pTRUST allows sensitive proteome characterization of a variety of bacterial endospores to aid in the search for protein biomarkers

**DOI:** 10.1371/journal.pone.0318186

**Published:** 2025-01-24

**Authors:** Masato Taoka, Ritsuko Kuwana, Yoshinari Murakami, Akiko Kashima, Yuko Nobe, Takamasa Uekita, Hiromu Takamatsu, Tohru Ichimura

**Affiliations:** 1 Department of Chemistry, Tokyo Metropolitan University, Tokyo, Japan; 2 Faculty of Pharmaceutical Science, Setsunan University, Osaka, Japan; 3 Department of Applied Chemistry, National Defense Academy, Kanagawa, Japan; 4 Carriere Reseau Co., Ltd., Kanagawa, Japan; DIU: Dhaka International University, BANGLADESH

## Abstract

Bacterial endospores are ubiquitous and are responsible for various human infections. Recently, we reported that an ionic liquid (IL)-based sample preparation method (named pTRUST) facilitated highly efficient shotgun analysis of the *Bacillus subtilis* spore proteome in trace samples. In this study, we evaluated the efficiency and applicability of the pTRUST technology using three different spore preparations: one purified from the closely related subspecies *B*. *subtilis* natto and two from *B*. *licheniformis* and *B*. *cereus*. We showed that the pTRUST method allowed rapid solubilization and processing of all tested spore samples prepared for highly sensitive mass spectrometry (MS) analysis. Bioinformatics analysis using the BLAST program suggested that a set of 25 proteins commonly identified between the above three species and *B*. *subtilis* spores may be universal biomarkers among various bacterial species, including 43 spore-producing bacteria associated with industrial dairy processing environments and product spoilage. In contrast, the two identified proteins, D4FV94 in *B*. *subtilis* natto and Q737A2 in *B*. *cereus*, are likely species-specific biomarkers, because their orthologs are absent or rare in all organisms. The sensitivity and applicability of pTRUST, along with the putative protein biomarkers identified in this study, will facilitate a wide spectrum of spore research for biological and clinical applications.

## Introduction

Bacteria normally multiply by repeated equal divisions, a process known as trophic propagation. However, certain bacteria, including those of *Bacillus* and *Clostridium* groups, undergo unequal division during nutrient starvation and form specialized structures called endospores (hereafter referred to as spores). Spores are a dormant form of bacteria and are among the organism’s structures that show the greatest resistance to physical and chemical insults [1]. Various spore-producing bacteria are pathogenic. For example, *Bacillus cereus* and *Bacillus weihenstephanensis* cause food poisoning, whereas *Bacillus anthracis* and *Clostridium botulinum* have been used in bioterrorism. Therefore, establishing rapid and sensitive detection systems for analyzing spore samples is essential for ensuring protection against these pathogens and diseases [2].

Spores are primarily composed of protein, DNA, and small molecules. Proteins are crucial for the formation, resistance, and pathogenicity of spore-forming bacteria [3]. Detailed knowledge of protein targets or protein biomarkers would thus aid in better understanding the molecular mechanisms of these biological processes and improve existing and emerging spore-based detection techniques to guarantee food and consumer safety. Various proteomic approaches have been developed to identify these molecules, primarily using non-pathogenic *B*. *subtilis* spores as models [4–8]. However, published procedures typically use conventional solubilizers, such as sodium dodecyl sulfate (SDS) and urea, to dissolve spore molecules, but the resistance of spore structures to these traditional agents makes it difficult to efficiently analyze proteins. Electron microscopic analysis has revealed that incubation of *B*. *subtilis* spores in SDS only disrupts some regions of the spores (for example, the coat and outer membrane), whereas most of the remaining regions (e.g., cortex, inner membrane, and core) remain visible [9]. Ultimately, large amounts of protein (20–800 μg) are typically required for these proteomic procedures [4–8], which are labor-intensive and time-consuming. Such large-scale preparations may also reduce the purity of spore samples and increase the risk of pathogenic transmission.

Ionic liquids (ILs) are powerful solvent media in biomedical and pharmaceutical applications [10]. We recently reported that *i*-soln (a mixture of the imidazolium-based IL, 1-butyl-3-methylimidazolium cyanate [bmim][SCN], and NaOH) can completely dissolve highly insoluble heat-aggregated hen egg whites within 10 min [11]. We also developed novel proteomic sample preparation methods (namely pTRUST [12] and its original version, *i*BOPs [11]) for direct processing of *i*-soln-solubilized samples with trypsin using hydrophobic microbeads. The analytical performance of these methods involving the *i*-soln system allowed the simple and sensitive proteomic characterization of various insoluble samples, including SDS-resistant aggregates deposited in senescent *Caenorhabditis elegans* and inclusion bodies [11,13], in addition to integral membrane proteins from various human cancer cell lines [12]. Very recently, we applied pTRUST to the spore proteome of *B*. *subtilis* and demonstrated that highly efficient shotgun analysis of the spore proteome was achieved even with micrograms or less of the starting material [14]. The analytical range observed for pTRUST was 50- to 2,000-fold higher than that previously reported for gel-based or gel-free approaches [4–8]. However, despite the superiority of this method in analyzing insoluble substances, its application in spore proteomics has been limited to the identification and characterization of resistance proteins in *B*. *subtilis* spores [14].

In this study, we evaluated the efficacy and generality of the pTRUST technology using highly purified spores from three spore-forming bacteria other than *B*. *subtilis*. We also analyzed the protein targets identified by pTRUST and mass spectrometry (MS), using a bioinformatics program to search potential spore biomarkers.

## Materials and methods

### Strains and materials

The *Bacillus* strains used in this study were *B*. *subtilis* subsp. natto BEST195, *B*. *licheniformis* ATCC 14580, and *B*. *cereus* ATCC 10987. Each of these strains produce spores, and their genomic sequences have already been determined. [bmim][SCN] was purchased from Sigma-Aldrich Co. LLC (St. Louis, MO, USA). *i*-soln was prepared by mixing [bmim][SCN] and 0.5 M NaOH (in water) at a 40:60 (v/v) ratio [11]. POROS R2 microbeads (diameter, 50 μm) were obtained from PerSeptive Biosystems, Inc. (Framingham, MA, USA). Before use, the beads (500 μg) were rinsed with 100 μL of 75% acetonitrile (CH_3_CN) in 0.1% trifluoroacetic acid and 100 μL of 100 mM Tris-HCl (pH 8) and suspended in 200 μL of water [14]. StageTips (polystyrene-divinylbenzene copolymer) was obtained from Nikkyo Technos Co., Ltd. (Bunkyo-ku, Tokyo, Japan). Other materials were purchased as previously described [11,12].

### Preparation and purification of spores

Bacteria were grown in Schaeffer’s medium at 37°C as previously described [4]. Spores were harvested 18 h after the cessation of exponential growth, washed in deionized water for several days, and collected upon centrifugation at 12,000 × *g* for 4 min at 4°C [4]. To purify the spores, the resultant pellets were incubated in 0.1 mL lysozyme buffer (10 mM Tris-HCl, pH 7.2, with 1% [w/v] lysozyme) for 10 min at 37°C and washed repeatedly with 10 mM Tris-HCl (pH 7.2) and 0.5 M NaCl at 25°C. More than 99% phase-bright spores and almost no dark or gray spores were obtained for all three bacterial samples, as assessed using phase-contrast microscopy [4]. The spores thus purified were resuspended in 10 mM Tris-HCl (pH 7.2) and frozen at -80°C. The purified spores were counted using colony formation assays on agar plates, as described previously [14]. The protein concentrations of the spores were determined using the Bradford assay [15], with bovine serum albumin as the standard.

### Spore lysis assay

To lyse the spores (2–4 × 10^8^ cfu), 1 mL *i*-soln was added and the mixture was incubated at 20°C using three cycles of ultrasonication (2 min) and agitation (1 min) in a water bath sonicator (ASU-10D; AS ONE Corporation, Osaka, Japan) and a vortex mixer, respectively [14]. Control experiments were performed in 1 mL water with or without sonication or 1 mL of 1% SDS with boiling for 3 min. The dissolution efficiency was assessed by measuring the turbidity value of the resulting solution at 600 nm using a UV-vis spectrophotometer (SmartSpecTM Plus; Bio-Rad Laboratories, Inc., Hercules, CA, USA).

### MS sample preparation using pTRUST

MS samples were prepared according to the previously defined pTRUST protocol [14]. For reduction of disulfide bridges, purified spores (5–6 × 10^6^ cfu each) containing 1 μg protein were incubated with 20 mM Tris(2-carboxyethyl)phosphine in 50 μL *i*-soln using three cycles of ultrasonication (2 min) and agitation (1 min) at 20°C. The reduced samples were treated with 40 mM iodoacetamide in the dark for 20 min to alkylate the free cysteines. Subsequently, the samples were mixed with the above R2 suspension, agitated for 1 min with a vortex mixer, and then allowed to stand for 1 min for protein adsorption onto the beads. After repeating the adsorption step four times, the bead–protein mixture was pipetted into a StageTip container and centrifuged at 2,000 × *g* for 30 s at 20°C to remove any excess *i*-soln from the beads that were retained on the StageTip filter [12,14]. The retained beads were sequentially washed with 100 μL Tris buffer (100 mM Tris-HCl, pH 8.0), 100 μL acetone twice, 100 μL Tris buffer, and 100 μL water via centrifugation under the same conditions. Trypsin digestion was performed at 37°C overnight with 0.5 μg trypsin in 20 μL trypsin digestion buffer (5 mM Tris, 60% CH_3_CN, pH 8.8) in a sealed StageTip container with rotation [12,14].

### LC-MS/MS analysis and protein identification

The peptide samples were recovered from the beads using centrifugation and underwent LC-MS/MS analysis as described [12,14]. The MS/MS data were converted into the Mascot-compatible data format using Proteome Discoverer (version 3.0; Thermo Fisher Scientific K.K., Tokyo, Japan) and the database search was performed using Mascot software (version 2.3.02; Matrix Science K.K., Tokyo, Japan) against UniProt *B*. *subtilis* subsp. natto BEST195 (taxid:645657), *B*. *licheniformis* strain ATCC 14580 (taxid:279010), and *B*. *cereus* strain ATCC 10987 (taxid:222523) proteome databases. The search parameters are the same as previously described: fixed modification for carbamidomethyl (C), variable modifications for acetylation (protein N-terminus) and oxidation (Met), maximum missed cleavage at 1, peptide mass tolerance of ±25 ppm, and MS/MS tolerance of ±0.8 Da [14]. Peptide identification threshold was based on the Mascot score p<0.05, which is commonly used and was validated in practice by our previous works [11–14].

### BLAST search

All BLAST searches were performed on the servers of the National Center for Biotechnology Information (NCBI). The identified proteins were searched against the NCBI non-redundant *B*. *subtilis* protein sequence database (*Bacillus subtilis* subsp. subtilis 168 [taxid:224308]) using the NCBI protein BLAST tool (https://blast.ncbi.nlm.nih.gov/Blast.cgi) version 2.15.0+ with preset algorithm parameters. Only sequences with >50% amino acid sequence identity and >60% alignment of protein sequences were considered putative orthologs of corresponding *B*. *subtilis* proteins. Some of the identified proteins (indicated in the text) were also searched against the corresponding NCBI non-redundant bacterial and whole-organism protein databases using the BLAST tool.

## Results

### Lysis of distinct bacterial spores with *i*-soln

**Fig 1:**
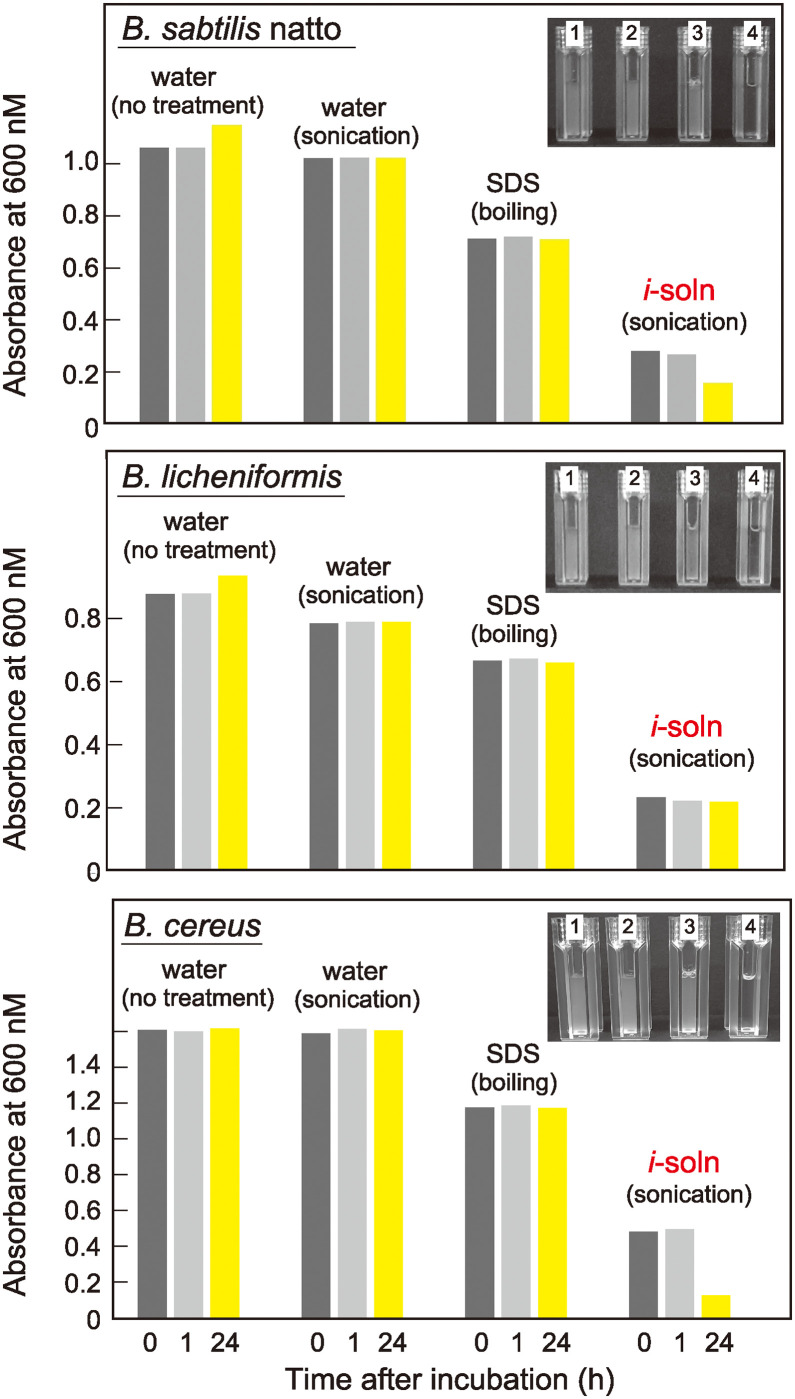
*i*-soln promotes the lysis of various bacterial spores. Purified spores were incubated in water and *i*-soln with or without sonication or boiled in 1% SDS as indicated. The turbidity of the solutions was analyzed at an absorbance of 600 nm with a UV–vis spectrophotometer after 0, 1, and 24 h. The mean absorbance values from each duplicate experiment are shown. The inset shows the solution images after 24 h: 1. water (no treatment), 2. water (sonication), 3. SDS (boiling), 4. *i*-soln (sonication).

We recently reported that *i*-soln can lyse *B*. *subtilis* (strain 168) spores with high efficiency by sonication only [14]. To determine whether *i*-soln is also effective for dissolving different bacterial spores, highly purified spores from the closely related subspecies *B*. *subtilis* subsp. natto (BEST195 strain) and two other species, *B*. *licheniformis* (ATCC 14580 strain) and *B*. *cereu*s (ATCC 10987 strain), were incubated in *i*-soln. As demonstrated in Fig 1, *i*-soln showed the highest dissolution efficiency at all time points in all samples compared with the controls suspended in water (with no treatment), sonicated in water as mentioned above, or boiled in 1% SDS, as assessed based on the turbidity (OD_600 nm_) of the resulting solution. In particular, the values in *i*-soln were approximately 20–30% of the no-treatment control values in all samples, even after 0 h. These values are consistent with those reported for *B*. *subtilis* spores [14]. Thus, *i*-soln can be applied directly to efficiently dissolve these bacterial spores.

### Proteomic identification and characterization of three *Bacillus* spores using pTRUST and LC-MS/MS

**Fig 2:**
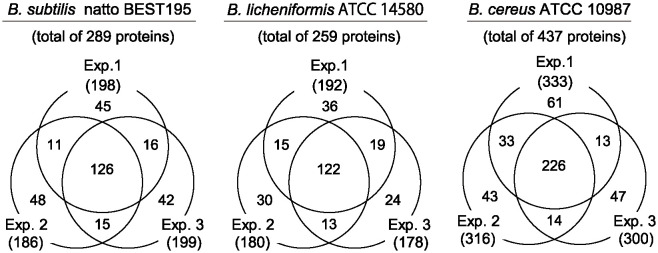
High overlapping rate of proteins identified in three pTRUST-MS runs of each spore preparation shown in Venn diagram.

To evaluate the efficiency and applicability of the present pTRUST method, purified spores solubilized with *i*-soln (each 1 μg protein) were processed with pTRUST in triplicates, and the resulting polypeptides were analyzed using LC-MS/MS. As shown in Fig 2, approximately 180–200 (for *B*. *subtilis* natto and *B*. *licheniformis*) and 300 (for *B*. *cereus*) proteins were consistently identified in each MS run, with good repeatability in the present analysis, even at the low-level (1 μg) quantity (see also S1–S3 Tables).

To characterize the identified proteins, we merged all identifications from each sample (a total of 289, 259, and 437 proteins from the spores of *B*. *subtilis* natto, *B*. *licheniformis*, and *B*. *cereus*, respectively, S1–S3 Tables) and analyzed their amino acid sequences using the UniProt protein database for molecular weight, isoelectric point, and GRAVY (grand average of hydropathicity) value. This analysis revealed a wide variety of biochemical properties attributable to the identified proteins (S4–S6 Tables), which were considered unbiased identifications using pTRUST for these parameters, as previously described [12,14]. Furthermore, the identified proteins included many known sporulation-related factors, such as those for spore coat Cot proteins, germination-associated Ger proteins, and a number of ribosomal subunits (S4–S6 Tables). Thus, the pTRUST method with the *i*-soln system efficiently processed these spore preparations for sensitive MS analysis, as reported for *B*. *subtilis* spores [14].

### Identification of putative protein biomarkers for detecting various or specific spores using BLAST search

**Fig 3:**
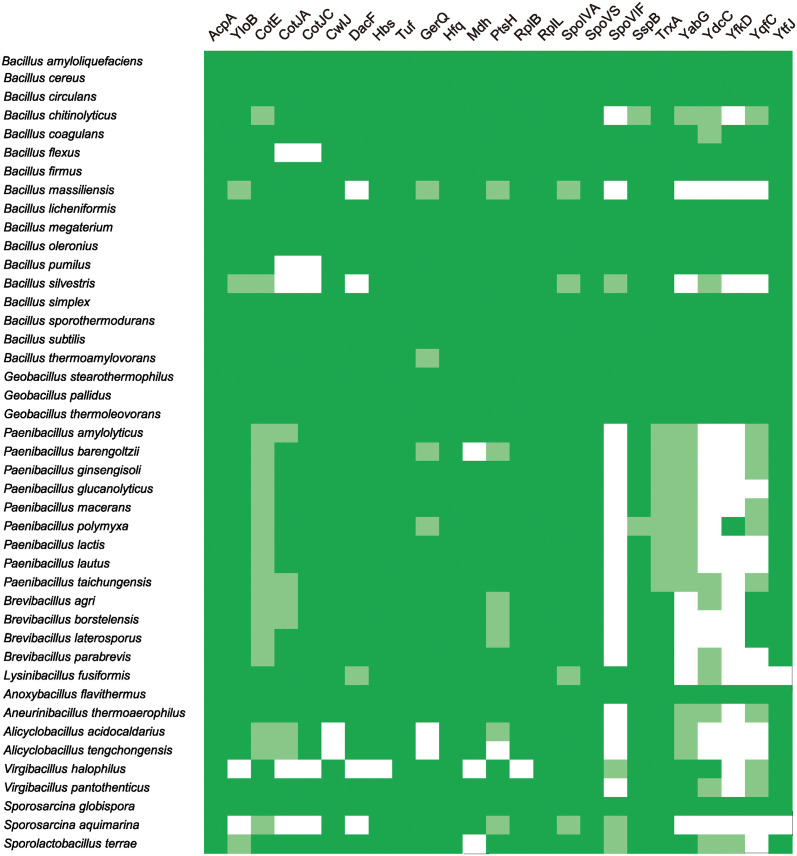
Widespread distribution of potential orthologous of the 25 identified proteins in 43 spore-forming bacteria associated with dairy processing and products. Dark-green boxes indicate proteins that are considered to be orthologous, based on a high level of amino acid sequence identity (>50%) and >60% alignment of the protein sequence. Light-green boxes indicate the proteins with amino acid sequence identity of 50–40% and >60% alignment of the protein sequence. White boxes indicate proteins with less than 39% amino acid sequence identity. See S8 Table for details.

To further characterize the identified proteins, their amino acid sequences were compared with those of the NCBI *B*. *subtilis* (strain 168) protein database using the BLAST search program. In relation to the phylogenetic distance between these *Bacillus* species [16], 231 (93.8%), 200 (77.2%), and 221 (49.0%) of the total proteins identified from the *B*. *subtilis* natto, *B*. *licheniformis*, and *B*. *cereus* spores, respectively, showed strong sequence identity with >50% of the corresponding *B*. *subtilis* proteins, even with >60% alignments of the protein sequences (S4–S6 Tables, marked in yellow). The high degree of conservation suggests that these are orthologous proteins that may have similar biological functions. Of these orthologs, a set of 25 proteins, comprising 14 of the sporulation-related proteins (CotE, CotJA, CotJC, CwlJ, DacF, GerQ, SpoIVA, SpoVS, SpoVIF, SspB, YabG, YdcC, YloB, and YqfC) (annotated in the SubtiWiki database), and 2 proteins involved in metabolism (AcpA and Mdh), 2 in DNA/RNA binding (Hbs and Hfq), 3 in protein translation (Tfu, RL2, and RL7), 2 in transport (PthP and YfkD), 1 in stress response (TrxA), and 2 uncharacterized proteins (YkfD and YtfJ), were common to all of the aforementioned spores, and also to the *B*. *subtilis* spores shown in a previous study [14] and in our recent unpublished work (for PtsH and YtfJ) (S7 Table). Further comparative studies using the reported amino acid sequences from 43 spore-forming bacteria associated with industrial dairy processing environments and product spoilage [17] revealed that 16 of these bacteria shared the same orthologs with all the 25 selected proteins (>50% identity) and that 42 bacteria shared 15 or more proteins (except for *Sporosarcina aquimarina*, 12 proteins) using the BLAST tool (Fig 3, S8 Table). We also found that three of the four proteins (CotJC, DacF, and SpoIVA) common among *B*. *subtilis* 168, *Clostridium difficile* 630, and *B*. *cereus* 14579 spores [18] are included in our list. Thus, the set of 25 proteins identified by pTRUST and LC-MS/MS represent the most likely universal biomarkers for detecting spores in various samples.

**Fig 4:**
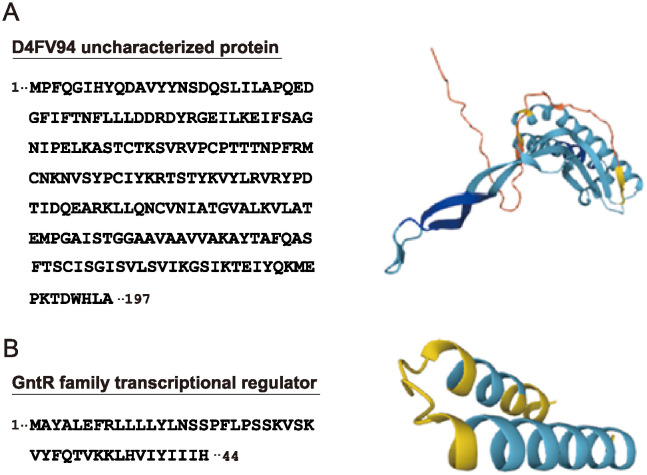
Primary sequence and 3D structure information of D4FV94 protein in *B*. *subtilis* natto (A) and GntR family transcriptional Q737A2 regulator in *B*. *cereus* (B). Data were taken from the UniProt database (Accession No. D4FV94_BACNB, Q737A2_BACC1).

Using BLAST, we then compared proteins with no sequence homology (0% identity) with *B*. *subtilis* (strain 168) proteins (161 proteins in total; see S4–S6 Tables) with those in the NCBI whole-organism protein database. We confirmed that, despite the lack of orthologs in the *B*. *subtilis* strain, many other bacterial species shared orthologous proteins (>50% identity) with the corresponding 161 proteins. Among these, at least nine proteins of *B*. *subtilis* natto (Accession No., A0A060PFM3, A0A060PFU1, A0A060PPB5, A0A060PPK7, D4FX13, D4FX61, D4FZQ5, D4G6U3, D4G799; S4 Table) appear to be the products of horizontal gene transfer (HGT) [19], because these orthologs are not present in the same species *B*. *subtilis* (strain 168). However, only two proteins did not meet the above criteria. One was a D4FV94 protein in *B*. *subtilis* natto (Accession No. D4FV94_BACNB), and the other was a GntR family transcriptional Q737A2 regulator in *B*. *cereus* (Accession No. Q737A2_BACC1) (Fig 4). There are no known functions for these two proteins. However, only a very few number of bacterial hypothetical proteins in the databases (e.g., that from *B*. *safensis*, *B*. *pumilus*, and *C*. *algoriphilum*) showed weak sequence similarity to the DAFV94 protein (<38% identity). In contrast, no proteins homologous to the transcriptional Q737A2 regulator in *B*. *cereus* were found in the whole-organism database. Thus, the two proteins identified in this study may be species-specific spore biomarkers whose orthologs are absent or rare in all organisms.

## Discussion

Proteomic analysis of spore samples remains a major challenge, owing to poor solubilization and extraction yields. In the present study, we showed that pTRUST and LC-MS/MS facilitated the rapid solubilization and processing of multiple proteins, including those characterized and uncharacterized previously, from trace amounts of purified spore preparations (S1–S3 Tables). To the best of our knowledge, this study is the first report on the proteomic characterization of *B*. *subtilis* natto and *B*. *licheniformis* spores and the first description of their proteomes directly associated with purified preparations (although there are reported cases of *B*. *cereus* spores [8,18]). These results support the expanded use of pTRUST in spore proteomics, which has so far been limited to the identification and characterization of resistance proteins in *B*. *subtilis* spores [14].

The pTRUST method has several advantages for spore analysis. (*i*) *i*-soln can dissolve spores more effectively than conventional solubilizers such as SDS (Fig 1), improving the efficiency of sample preparation for high-spec MS analysis. (*ii*) pTRUST is simple and does not require the additional sample purification steps necessary in previous methods such as PAGE or hydrophobic chromatography [4–8]. (*iii*) pTRUST enables efficient processing of a variety of low-abundance (or low-concentration) spore samples (Fig 2, S1–S3 Tables). Indeed, the pTRUST protocol using hydrophobic R2 bead supports can quantitatively capture most proteins with no selectivity and enhance the catalytic activity of trypsin and solubility of tryptic peptides during the digestion reaction [12,14]. Furthermore, the small-sized StageTip container used for trypsin digestion can stimulate small-scale enzymatic digestion (<20 μL) by decreasing the surface area involved in non-specific adsorption losses of proteolytic peptides [12,14]. Therefore, we propose pTRUST as one of the simplest and most practical platforms for characterizing spore proteins and biomarkers that are otherwise difficult to detect because of their low abundance.

The direct detection of spores is critical for determining microbial contamination in various types of food and environmental samples and for protecting against natural infections and biological threats [20]. Various techniques targeting spore nucleic acids, metabolites (such as dipicolinic acid and ATP), and proteins have been exploited to detect spores, but each of the traditional methods has several shortcomings, especially those involving their stability [21,22]; thus, the discovery and characterization of new targets is expected. In this regard, one notable trend in the present study is that many proteins identified in the purified spore samples have orthologs between the used species as well as other bacteria (Fig 3, S4–S8 Tables). Such factors may play key roles in general spore physiology, including sporulation, germination and outgrowth to vegetative cells. In contrast, the D4FV94 protein and GntR family transcriptional Q737A2 regulator are rare or absent in the organism-wide database (Fig 4). Although their functions have not been characterized, they may be involved in species-specific spore phenomena.

We previously produced green fluorescent protein (GFP) fusions from 20 newly identified *B*. *subtilis* proteins and demonstrated, using fluorescence microscopy, that all these candidates were authentic spore components [14]. Validating proteomic data using such an alternative approach can effectively corroborate the accuracy and reliability of identification. Therefore, for a more in-depth evaluation, similar cell-imaging assays using GFP will be necessary to test the validity of the identified proteins and candidate biomarkers.

In conclusion, the pTRUST method involving the *i*-soln system allowed us to identify various previously uncharacterized proteins and potential biomarkers that may be associated with spores. The pTRUST technology improves upon other current approaches and is likely to be useful as a general procedure for sensitive spore characterization at the protein level. The pTRUST protocol is rapid, with a full cycle time of only 45 min before trypsin digestion. When used in combination with conventional quantitative techniques such as stable isotope labeling [23] and label-free methods [24], this technology can also be adapted without modification to sensitive spore-protein dynamic analysis. We believe therefore that pTRUST opens new avenue of investigation for a wide range of biological and therapeutic applications in spore research.

## Supporting information

S1 Table Proteins identified using pTRUST and liquid chromatography with tandem mass spectrometry (LC-MS/MS) in highly purified *Bacillus subtilis* subsp. natto spores. (XLSX)

S2 Table Proteins identified using pTRUST and LC-MS/MS in highly purified *Bacillus licheniformis* spores. (XLSX)

S3 Table Proteins identified using pTRUST and LC-MS/MS in highly purified *Bacillus cereus* spores. (XLSX)

S4 Table Characterization of proteins identified using pTRUST and LC-MS/MS in highly purified *Bacillus subtilis* subsp. natto spores. (XLSX)

S5 Table Characterization of proteins identified using pTRUST and LC-MS/MS in highly purified *Bacillus licheniformis* spores. (XLSX)

S6 Table Characterization of proteins identified using pTRUST and LC-MS/MS in highly purified *Bacillus cereus* spores. (XLSX)

S7 Table Potential orthologous proteins identified in all four *Bacillus* spores. (XLSX)

S8 Table Potential orthologous proteins found in the 43 spore-producing bacteria associated with dairy processing and products. (XLSX)
